# Pathological networks and multi-target interventions in sepsis-associated acute lung injury: from pathogen-host interactions to gut-lung axis regulation

**DOI:** 10.3389/fimmu.2026.1858653

**Published:** 2026-09-07

**Authors:** Jinying Sui, Min Chen, Lijie Qiu, Biao Ni, Shaoqiang Wang, Bo Liu

**Affiliations:** 1 School of Clinical Medicine, Shandong Second Medical University, Weifang, Shandong, China; 2 Department of Respiratory Medicine, The First Affiliated Hospital of Shandong Second Medical University (Weifang People’s Hospital), Weifang, Shandong, China; 3 Research Management Department, The First Affiliated Hospital of Shandong Second Medical University (Weifang People’s Hospital), Weifang, Shandong, China

**Keywords:** acute respiratory distress syndrome, ferroptosis, gut-lung axis, pathogen-host interaction, precision medicine, sepsis

## Abstract

**Objective:**

Sepsis is a life-threatening organ dysfunction resulting from a dysregulated host response to infection, accounting for an estimated 11 million deaths annually worldwide. Among its most prevalent and severe complications is acute respiratory distress syndrome (ARDS). Traditional research has focused on isolated pathways such as inflammation and oxidative stress; however, the results of clinical trials for related targeted drugs have been less than ideal. The core reason is that sepsis-associated acute lung injury (SA-ALI) is not driven by a single, independent pathway, but rather by an interconnected pathological network activated jointly by pathogen-associated molecular patterns (PAMPs) and damage-associated molecular patterns (DAMPs), featuring multiple positive feedback loops. In this network, pathogen virulence factors serve as upstream initiation nodes, while mitochondrial dysfunction acts as a central regulatory hub. Downstream, four major effect modules—immune and inflammatory dysregulation, oxidative stress and coagulation imbalance, bidirectional disruption of the gut-lung axis, and ferroptosis—are linked in series. Multiple links in this network synergistically amplify damage to alveolar epithelium and endothelium, ultimately leading to diffuse pulmonary edema, gas exchange failure, and multi-organ failure.

**Methods:**

This paper systematically searched PubMed, Web of Science, and CNKI databases from 2000 to 2026, using the search terms “sepsis, acute lung injury, ferroptosis, gut-lung axis, phage therapy, and mitochondrial-targeted interventions.” We screened for literature on fundamental mechanisms, animal studies, clinical cohort studies, and reviews, while excluding low-relevance studies based solely on *in vitro* cellular validation or lacking *in vivo* or clinical sample support. Throughout the paper, a three-tier interconnected pathological network serves as the unifying framework. We systematically analyze the interaction patterns between pathogenic microorganisms and the host’s immune and metabolic networks, compare the distinct damage pathways induced by virulence factors of different pathogens, and elucidate, layer by layer, the cross-talk among the immune-inflammatory, oxidative stress, gut-lung axis, and ferroptosis modules; At the same time, it categorizes all emerging therapies by stage, conducts critical evaluations based on their clinical translation status, and constructs a phased, sequential, multi-target synergistic treatment framework.

**Results:**

1. The virulence pathways of different bacteria, fungi, and viruses exhibit a pattern of “heterogeneous triggering and downstream convergence,” with damage signals from various pathogens ultimately converging at two core network hubs: mitochondrial dysfunction and ferroptosis; 2. There are multilevel positive feedback loops among immune and inflammatory dysregulation, oxidative stress, coagulation disorders, the gut-lung axis, and ferroptosis; blocking a single pathway is offset by compensatory pathways within the network, which explains the clinical failure of single-target drugs; 3. Existing interventions can be classified into five major categories: upstream anti-infection, midstream mitochondrial protection, downstream inhibition of inflammation/ferroptosis, epigenetic regulation, and natural small-molecule adjuvants. Each therapeutic approach has well-defined synergistic targets, and their combined use can simultaneously block multiple damage nodes within the network; 4. The source of infection, age, immune status, and underlying comorbidities reshape the topological structure of the pathological network, leading to heterogeneity in treatment response and necessitating stratified, individualized interventions.

**Discussion:**

This review overcomes the limitations of traditional reviews—which often present “fragmented discussions focused on a single pathway”—by using an interconnected pathological network as the central thread to link all content throughout. It innovatively proposes a three-tiered network regulation theory; systematically compares the development stages, clinical bottlenecks, and synergistic value of various therapies; and provides a comprehensive theoretical framework for precision stratified treatment of SA-ALI and the identification of new drug targets. Targeting central network nodes such as mitochondria and employing phased, sequential multi-target combination regimens offer superior lung-protective effects compared to single-pathway inhibitors and represent a core research direction for improving the long-term prognosis of patients with sepsis complicated by ARDS.

## 1 Introduction

Sepsis presents a major global health challenge, characterized by a life-threatening, dysregulated host response to infection that leads to widespread tissue injury and multi-organ dysfunction. Acute lung injury (ALI) and its severe form, ARDS, are among the most critical complications (1–4). Epidemiological studies underscore its severity, with an overall mortality rate of approximately 20% in the general population, rising to 33% in high-risk groups (5–7). A large-scale study in Chinese intensive care units found that ALI develops in up to 68.2% of septic patients, and progression to ARDS is associated with a 90-day mortality rate of 35.5% (8), highlighting the dire prognosis. Survivors often face significant long-term sequelae, including persistent immune dysfunction and muscle atrophy, requiring extended care (9, 10). Within this complex pathophysiology, the lungs are particularly vulnerable, frequently serving as the first and most severely affected organ.

The pathogenesis of sepsis-associated ALI (SA-ALI) is multifactorial, driven by a dysregulated interplay between pathogenic microorganisms and the host’s immune-metabolic responses. Traditional research and therapeutic approaches have predominantly targeted singular pathways, such as excessive cytokine production or oxidative stress (11); however, emerging evidence suggests a more complex and integrated pathogenic network. This network encompasses synergistic interactions between specific virulence factors of principal pathogens (e.g., *Streptococcus pneumoniae*, *Klebsiella pneumoniae*, *Pseudomonas aeruginosa*) and host defense mechanisms, including immune-inflammatory dysregulation, coagulation disturbances, disruption of the gut-lung axis, and novel programmed cell death modalities such as ferroptosis (11–14). Concurrently, the escalating prevalence of antimicrobial resistance among these key pathogens further compromises conventional anti-infective therapies, thereby necessitating the development of innovative therapeutic strategies that extend beyond direct pathogen eradication (15).

Despite these advances, current research predominantly addresses individual pathogens or isolated pathogenic mechanisms, resulting in a fragmented understanding. A critical unaddressed gap is that most existing reviews describe inflammatory dysregulation, ferroptosis, and gut-lung axis crosstalk as independent pathways, failing to recognize that SA-ALI is essentially a compensatory pathological network with robust positive feedback loops—this very network architecture explains the consistent failure of single-target therapies in clinical trials (16). The intrinsic relationships and synergistic interactions among these mechanisms remain insufficiently characterized, particularly due to the absence of an integrated analytical framework capable of synthesizing pathogen-specific attributes with multidimensional host responses (7, 17). To fill this gap, this review proposes a novel three-tiered pathological network framework: upstream trigger nodes (pathogen virulence factors), central regulatory hub (mitochondrial dysfunction), and downstream effector modules (immune inflammation, oxidative stress-coagulopathy, gut-lung axis, and ferroptosis). Moreover, multiple therapeutic targets have been identified across different stages of disease progression, the integration of multi-target intervention strategies into coordinated, host-directed treatment regimens remain a significant challenge for clinical application. Building on this network perspective, we further propose a sequential multi-target synergistic therapeutic strategy tailored to disease stages, rather than a simple compilation of single-pathway interventions. Such approaches must concurrently achieve effective pathogen clearance, precise modulation of immune responses, and prevention of secondary tissue injury (2).

To address this challenge, this review aims to systematically construct an integrated framework encompassing pathogen, host, and therapeutic perspectives. Firstly, it elucidates the pathogenic mechanisms of key pathogens, revealing their interactions with host immune-metabolic networks (12). Following this, it offers a comprehensive analysis of the interplay among fundamental processes, including immune-inflammatory dysregulation, oxidative stress, coagulation abnormalities, gut-lung axis interactions, and ferroptosis (11–14). Building on this foundation, the review critically appraises a range of emerging therapeutic approaches targeting these pathways, such as optimized antibiotic regimens, phage therapy, mitochondrial-targeted treatments, epigenetic modulation, and bioactive natural compounds. Ultimately, the paper proposes a multi-layered, multi-targeted synergistic therapeutic strategy designed to provide a theoretical basis and strategic direction for advancing mechanistic research and precision clinical interventions in SA-ALI (18, 19).

## 2 Research methods

### 2.1 Search strategy

Databases: PubMed, Web of Science, CNKI, Wanfang; Search period: January 2000–June 2026. Chinese and English keywords included sepsis, ALI, ferroptosis, mitochondria, phages, and epigenetic regulation.

### 2.2 Inclusion/exclusion criteria

Inclusion: Studies involving *in vivo* animal models (mice and rats), human clinical cohorts, randomized controlled trials, and systematic reviews that provide data on pathway interactions; Exclusion: Studies limited to *in vitro* cellular models, those without statistical controls, duplicate publications, and short articles focusing solely on a single pathway without cross-analysis.

### 2.3 Construction of the review framework

The full text follows a three-tiered pathological network as its logical backbone, with a summary of network interactions added at the end of each subsection to clarify the upstream and downstream interactions of that module within the overall damage pathway.

## 3 Virulence factors of pathogens and host interactive network mechanisms in sepsis-associated acute lung injury

From the perspective of the integrated pathological network, diverse pathogenic microorganisms act as the upstream triggers of sepsis-associated acute lung injury (SA-ALI). This section systematically summarizes the key virulence factors of bacteria, opportunistic fungi and viruses, as well as their corresponding lung injury pathways. All pathogens share a unified pathological feature: they exert distinct upstream virulent effects yet converge on identical downstream injury cascades. *Streptococcus pneumoniae*, *Pseudomonas aeruginosa* and methicillin-resistant *Staphylococcus aureus* (MRSA) all disrupt vascular endothelial integrity and trigger the formation of neutrophil extracellular traps (NETs). However, they induce unique tissue damage via pore-forming toxins, matrix-degrading enzymes and α-hemolysin respectively. Ultimately, all microbial toxins initiate mitochondrial dysfunction and ferroptosis, giving rise to consistent downstream pathological cascades (20, 21).

Pathogens in this section are classified into three categories: common pathogenic bacteria, opportunistic fungi and viruses, and direct causal relationships between various toxins and the onset of acute lung injury are demonstrated. Fungi predominantly provoke pulmonary inflammation through yeast-hyphal morphological transformation, while viruses bind specific cell receptors to activate inflammatory signaling pathways and exacerbate subsequent tissue damage. The synergistic effects of multiple microbial virulence factors, combined with widespread bacterial antimicrobial resistance, limit the therapeutic efficacy of conventional single-agent anti-infective therapies. This further highlights the superior clinical value of combinatorial treatment regimens that simultaneously eliminate pathogenic bacteria and target virulence factors. This section systematically elaborates on the core virulence factors and injury pathways of bacteria, fungi, viruses associated with SA-ALI. Further analysis reveals a characteristic pattern of “common pathway anchoring and specific target differentiation” in lung injury induced by different pathogens: pathogenic bacteria such as *Streptococcus pneumoniae*, *Pseudomonas aeruginosa*, and methicillin-resistant *Staphylococcus aureus* all take disrupting the endothelial barrier and inducing neutrophil NETs formation as common injury pathways, but achieve specific pathogenicity through membrane pore formation, extracellular matrix degradation, and toxin-mediated oxidative stress, respectively. Notably, these distinct virulence factors ultimately converge on shared core host pathways, including mitochondrial dysfunction and ferroptosis, forming a network feature of “heterogeneous triggering converging to common downstream effectors” (22). Fungi and viruses trigger lung injury by superposing the common inflammatory activation pathway through their unique modes such as morphological transformation and receptor binding (23). This pathogenic mechanisms difference provides a core molecular basis for formulating precise anti-injury strategies according to the type of infectious pathogen, and also reveals the heterogeneous nature of the SA-ALI pathogen-host interaction network. At the same time, the evolution of pathogen resistance mechanisms and the synergistic effect of virulence factors have become the key reasons for the failure of traditional anti-infective therapy, highlighting the necessity of combined intervention of “targeting virulence factors and eliminating pathogens” (24).

### 3.1 Bacterial pathogens

Worldwide, the prevalence and incidence of infections caused by *Streptococcus pneumoniae* keep increasing; the bacterium is significantly involved in childhood pneumonia Annually, pneumococcal disease accounts for approximately one million fatalities among children under five years of age (25). For *Streptococcus pneumoniae*, pneumolysin, a pore-forming cytolysin, is a core virulence effector. Accumulating *in vivo* evidence from isogenic mutant studies consistently demonstrates that pneumolysin is indispensable for invasive pneumococcal disease. In murine infection models, pneumolysin-deficient strains exhibit severely impaired dissemination from the alveolar space into the bloodstream, reduced incidence of bacteremia and secondary meningitis, and significantly attenuated lethality. These findings establish a direct causal link between this toxin and the progression from localized pneumonia to systemic sepsis (26). At the tissue level, pneumolysin disrupts the integrity of alveolar epithelial and pulmonary endothelial barriers by forming transmembrane pores in host cell membranes, which facilitates bacterial translocation across the alveolar-capillary barrier and initiates early pulmonary inflammatory injury (27).

Beyond direct cytolytic damage to host cells, a recently identified mechanism further amplifies pneumolysin-mediated pathogenicity: sublytic concentrations of pneumolysin trigger human monocytes to shed extracellular vesicles (EVs) that carry membrane-bound toxin and enriched inflammatory proteins including IFI16, NLRC4, and MMP9. These toxin-loaded host EVs can deliver pneumolysin to distant recipient cells such as dendritic cells, induce recipient cell cytotoxicity, and propagate pro-inflammatory cascades. *In vivo* administration of these pneumolysin-induced EVs directly causes tissue damage, systemic inflammation and mortality in experimental models, indicating that this EV-mediated toxin diffusion pathway contributes to the amplification of local pulmonary injury and the development of remote organ dysfunction during sepsis (28). Concurrently, pneumolysin activates innate immune effectors such as macrophages, eliciting the secretion of pro-inflammatory cytokines including interleukin-1β (IL-1β) and tumor necrosis factor-alpha (TNF-α), thereby potentiating localized inflammatory responses (25, 29).

The polysaccharide capsule functions as a critical immune evasion structure, impeding complement activation and phagocytic recognition, while also inhibiting the effective formation of neutrophil extracellular traps (NETs). This multifaceted immune camouflage substantially impairs bacterial clearance, facilitating proliferation and systemic dissemination (25, 29, 30). Moreover, *S. pneumoniae* expresses various surface adhesins, such as pneumococcal surface adhesin A (PsaA) and choline-binding protein A (CbpA), which mediate adherence to specific receptors on the respiratory mucosa. This adherence enables nasopharyngeal colonization and, under conditions of host immune compromise, predisposes to invasive pathologies including pneumonia, otitis media, meningitis, and sepsis (29).

At the mechanistic intersection with the core host network, pneumolysin (PLY) pore formation triggers sustained calcium influx in alveolar epithelial cells, which initiates mitochondrial fragmentation and mitochondrial membrane depolarization (31). Mitochondrial fission driven by activated Dynamin-Related Protein 1 (Drp1) has been validated as an essential upstream event that consumes intracellular Glutathione (GSH), suppresses Glutathione Peroxidase 4 (GPX4) activity, and ultimately initiates epithelial ferroptosis (32). Moreover, the polysaccharide capsule of *S. pneumoniae* blocks macrophage autophagic clearance of damaged organelles, which exacerbates ferroptosis-derived inflammatory signal release.

*Klebsiella pneumoniae* (KP) is implicated in severe infections such as pneumonia, liver abscesses, and urinary tract infections. KP-associated pneumonia is characterized by a poor clinical prognosis, with mortality rates ranging from 30% to 50% despite optimal therapeutic interventions (33). Capsular polysaccharide (CPS) represents the predominant virulence factor. Hypervirulent KP strains exhibit markedly thickened capsules with heterogeneous polysaccharide compositions, facilitating complex immune evasion strategies (33, 34). The capsule physically obstructs macrophage-mediated phagocytosis, inhibits phagosome-lysosome fusion, and suppresses complement activation, thereby diminishing membrane attack complex (MAC) formation and bacterial lysis (34). Additionally, capsular polysaccharides can activate the nuclear factor kappa-light-chain-enhancer of activated B cells (NF-κB) signaling pathway, inducing excessive production of inflammatory cytokines such as interleukin-6 (IL-6) and TNF-α. This hyperinflammatory response precipitates pulmonary tissue consolidation, necrosis, and abscess formation, which are characteristic of the rapid progression to septic acute lung injury (33, 34).

With respect to network crosstalk, carbapenem-resistant hypervirulent *K. pneumoniae* (CR-hvKP) disrupts macrophage redox homeostasis by perturbing the SLC7A11/GSH antioxidant axis, altering intracellular iron storage and elevating lipid peroxidation burden; sustained iron overload in infected macrophages ultimately primes ferroptotic signaling and amplifies pulmonary inflammatory cascades, even though pharmacological inhibition of SLC7A11 can partially restore bactericidal function against CR-hvKP (35).

*Pseudomonas aeruginosa* accounts for approximately 10% of nosocomial infections and up to 17% of ventilator-associated pneumonia (VAP) cases, with associated mortality rates reaching 40% (36). Its pathogenicity is mediated by an array of secreted virulence factors. Elastase enzymatically degrades extracellular matrix components, including elastin and collagen within pulmonary tissue, thereby compromising alveolar structural integrity and inducing parenchymal necrosis and hemorrhage. Elastase also hydrolyzes immunoglobulins and complement proteins, attenuating host immune defenses (36, 37). The pigment pyocyanin, responsible for the characteristic green coloration of infected pus, catalyzes the generation of reactive oxygen species (ROS), inflicting direct damage to cellular macromolecules in alveolar cells. Concurrently, pyocyanin activates inflammatory signaling pathways, promoting the release of IL-1 and IL-6, thus establishing a deleterious cycle of oxidative stress and inflammation (37). Phospholipase C contributes to the degradation of pulmonary surfactant lipids, thereby facilitating alveolar collapse and the development of atelectasis. The synergistic effects of these pathological processes frequently present clinically with symptoms such as high fever, the expectoration of emerald-green purulent sputum, and a rapid progression to respiratory failure (36).

*Pseudomonas aeruginosa* employs multiple virulence factors to initiate epithelial ferroptosis cascade. Pyocyanin acts as a redox-cycling toxin that continuously depletes intracellular GSH pools and boosts cellular ROS generation, priming alveolar cells for lipid peroxidation (38). Meanwhile, the secreted bacterial lipoxygenase pLoxA directly oxidizes membrane polyunsaturated phosphatidylethanolamines and accelerates GPX4 degradation, while elastase B disrupts mitochondrial outer membrane integrity to trigger excessive fission; together these toxins create an irreversible oxidative injury positive feedback loop (39).

Methicillin-resistant *Staphylococcus aureus* (MRSA) demonstrates extensive resistance to commonly employed antimicrobial agents and exhibits significant adaptive capabilities, with initial treatment failure rates reported to reach as high as 41.6% (40). This pathogen secretes a variety of exotoxins, among which α-hemolysin (Hla) plays a pivotal role in mediating pulmonary injury. Hla induces pore formation in the membranes of alveolar epithelial and vascular endothelial cells, leading to cell death through osmotic lysis. Concurrently, Hla activates macrophages and neutrophils, inducing the release of large amounts of pro-inflammatory cytokines including IL-6, IL-8, and TNF-α, as well as reactive oxygen species (ROS). This cascade precipitates a pulmonary inflammatory storm characterized by extensive infiltration of inflammatory cells, alveolar edema, and increased capillary permeability (40, 41). In addition, MRSA employs multiple immune evasion mechanisms. Surface protein A (SpA) binds to the Fc region of immunoglobulin G (IgG), thereby inhibiting antibody-mediated opsonization and subsequent phagocytosis. The bacterium also secretes chemokine-inhibitory proteins that obstruct neutrophil recruitment to the site of infection, diminishing the efficiency of bacterial clearance (42). Although vancomycin remains a critical last-resort therapeutic agent, the emergence and increasing isolation of vancomycin-resistant strains in clinical settings have further constrained treatment options, highlighting the urgent necessity for the development of novel and effective therapeutic strategies (40, 43).

At the network level of injury, α-hemolysin forms transmembrane pores in vascular endothelial cells, triggering calcium influx and barrier disruption as a well-characterized cytotoxic effect of this major staphylococcal virulence factor (44). Mitochondrial fission and dysfunction, which are widely recognized as central downstream events of calcium overload and inflammatory injury in sepsis-associated acute lung injury, further propagate cellular damage (45). As mitochondrial dysfunction represents a core upstream driver of ferroptosis (46), this cascade creates a mechanistic link between staphylococcal virulence factors and ferroptotic cell death in the injured lung.

### 3.2 Fungi and viruses

*Candida albicans* primarily evades host immune clearance through its ability to undergo dimorphic switching between yeast and hyphal morphologies, as well as through the formation of biofilms (47). During initial colonization, *C. albicans* exists predominantly in the unicellular yeast form, which facilitates evasion of host immune defenses. Under favorable conditions, such as host immunosuppression or alterations in the local microenvironment, the organism rapidly transitions to the invasive hyphal form. The hyphae utilize apical structures, including germ tubes, to penetrate the respiratory epithelial barrier and invade deeper pulmonary tissues (47, 48).

The hyphal morphology is characterized by increased tissue invasiveness and the secretion of aspartyl proteases that degrade critical pulmonary structural proteins, such as collagen and elastin, as well as host immune components including antibodies and complement proteins. This enzymatic activity promotes dissemination of the infection. Concurrently, the hyphal form elicits a pronounced local inflammatory response, manifesting as alveolar edema, infiltration of inflammatory cells, and tissue necrosis (48). Additionally, C. albicans can acquire antifungal resistance through genetic mutations, for example in the ERG11 gene, and through the overexpression of efflux pumps encoded by genes such as CDR1 and CDR2. These resistance mechanisms complicate clinical management and contribute to ongoing inflammatory lung injury (47–49).

*C. albicans* represents the most prevalent species within the Candida genus, accounting for approximately 80% of nosocomial fungal infections. Such infections are often associated with poor therapeutic outcomes due to both antimicrobial resistance and adverse drug effects, frequently resulting in acute lung injury with high morbidity and mortality rates (49).

**Figure 1:**
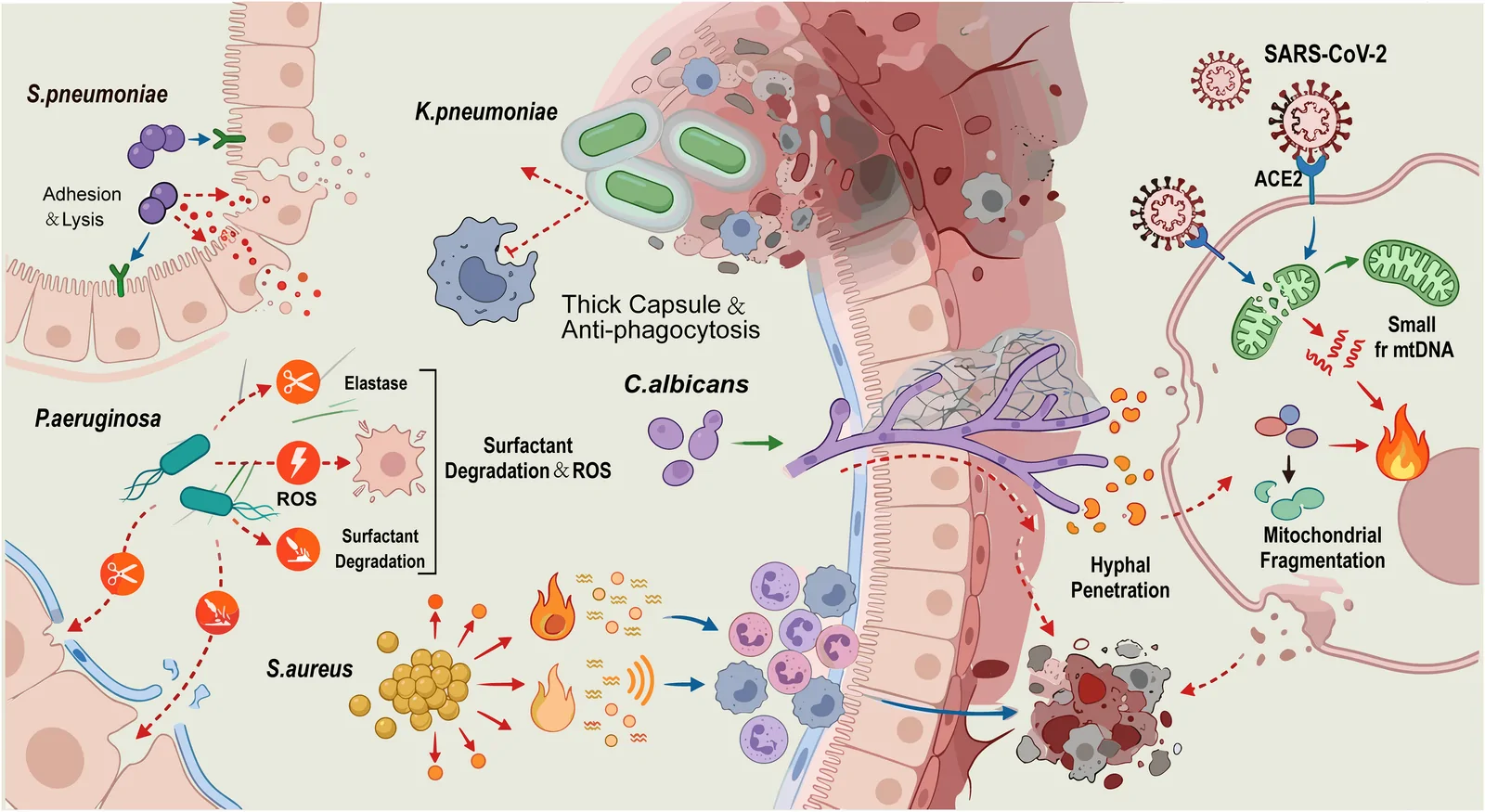
Pathogen-specific injury mechanisms underlying sepsis-associated acute lung injury (SA-ALI). This schematic systematically illustrates the core virulence cascades of six major pathogens responsible for septic lung injury, including *Streptococcus pneumoniae*, *Klebsiella pneumoniae*, *Pseudomonas aeruginosa*, MRSA, *Candida albicans*, and SARS-CoV-2. Each pathogen adopts unique pathogenic tactics: *S. pneumoniae* mediates alveolar epithelial lysis via adhesion proteins and pore-forming toxins; *K. pneumoniae* evades host immune clearance through thick anti-phagocytic capsules; dimorphic switching of *C. albicans* drives surfactant degradation, reactive oxygen species (ROS) accumulation and invasive hyphal penetration into pulmonary parenchyma. Both *P. aeruginosa* and *S. aureus* secrete elastase and excessive ROS to disrupt alveolar integrity, while SARS-CoV-2 binds alveolar ACE2 receptors to trigger mitochondrial fragmentation and amplify oxidative damage. Collectively, this diagram demonstrates that disparate virulence programs of diverse pathogens ultimately converge on shared downstream pathological events, including epithelial barrier breakdown, oxidative stress and systemic immune dysregulation, which supports the unified pathological network paradigm of “heterogeneous triggers converging to identical lung injury phenotypes” in sepsis.

Viral pathogens, particularly influenza viruses and SARS-CoV-2, are major contributors to sepsis-induced acute lung injury. SARS-CoV-2 achieves high-affinity binding to the host angiotensin-converting enzyme 2 (ACE2) receptor by means of its surface spike (S) protein. Infected individuals commonly present with significantly elevated systemic inflammatory markers, including serum IL-6, C-reactive protein (CRP), and ferritin, reflecting a severe inflammatory state (50, 51). In contrast, influenza viruses gain entry into host cells by binding to sialic acid receptors on respiratory epithelial cells through the haemagglutinin (HA) protein. The primary cellular targets include alveolar epithelial cells, vascular endothelial cells, and immune cells. Direct viral infection induces cellular dysfunction or death, leading to disruption of lung tissue architecture and impaired gas exchange (52, 53) (**Figure 1**).

## 4 Integrated architecture of the SA-ALI pathological network

The core pathological process of SA-ALI is not driven by any single mechanism acting independently, but constitutes a cascade amplification network with mitochondrial dysfunction as the central hub. This network connects multiple pathological modules, including immune-inflammatory dysregulation, oxidative stress-coagulopathy, bidirectional gut-lung axis regulation, and ferroptosis.

**Figure 2:**
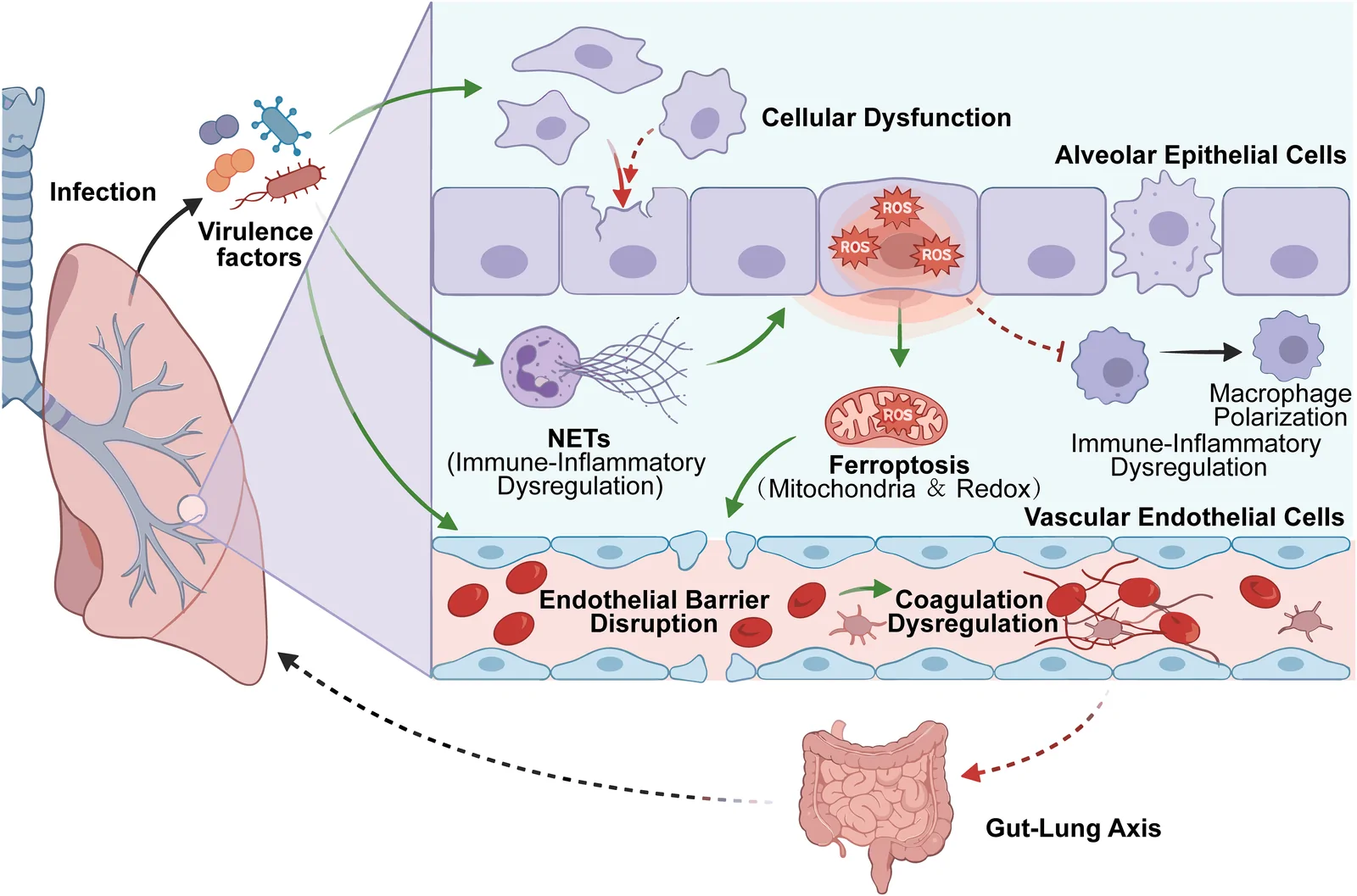
Integrated pathological cascade network of sepsis-induced acute lung injury. Centered on the three-tier pathological network framework proposed in this review, this image delineates the progressive injury cascade spanning initial infection to multi-organ crosstalk. Microbial virulence factors released at the upstream stage simultaneously provoke alveolar epithelial dysfunction and aberrant macrophage polarization, which further stimulate massive neutrophil extracellular trap (NET) formation and systemic immuno-inflammatory dysregulation. At the central regulatory hub, mitochondrial dysfunction and ferroptosis act as master amplifiers, exacerbating endothelial barrier disruption and coagulation disorders. Downstream, the gut-lung axis serves as a cross-organ amplifier: intestinal barrier impairment and microbiota dysbiosis reciprocally aggravate pulmonary inflammatory damage. This visualization confirms that SA-ALI is not driven by isolated signaling pathways, but arises from interconnected positive feedback loops linking immune disturbance, redox imbalance, coagulopathy, gut-lung communication and ferroptosis.

As the central hub of the entire network, mitochondrial fragmentation and dysfunction induced by pathogen invasion serve as the upstream source of massive ROS release and the core driver of lipid peroxidation in ferroptosis; meanwhile, mitochondrial abnormalities aggravate endothelial apoptosis and immune cell dysfunction via energy metabolism imbalance. The gut-lung axis acts as a cross-organ amplifier centered on “metabolism-immunity” crosstalk. Immune-inflammatory dysregulation functions as the core signal amplifier, linking upstream pathogen stimulation to multiple downstream effector pathways. Ferroptosis, as a terminal effector modality of cell death, forms positive feedback loops with NET formation and pyroptosis. The cascade relationship of upstream triggering, midstream amplification, and downstream effect clarifies the integrity and connectivity of the SA-ALI pathological network, and provides a rationale for screening core intervention targets that cover multiple mechanisms simultaneously. The following sections will detail the molecular mechanisms of each module and their crosstalk within the network framework (**Figure 2**).

### 4.1 Impact of infection source on sepsis-induced lung injury

The anatomical source of infection significantly shapes the pathological trajectory and mechanistic features of sepsis-induced lung injury, as different pathogen entry routes trigger distinct systemic immune and inflammatory patterns.

Pulmonary-origin sepsis (e.g., community-acquired pneumonia, ventilator-associated pneumonia) causes direct primary lung injury via local bacterial invasion and toxin release, with early and prominent alveolar epithelial damage and alveolar edema. In contrast, extrapulmonary sepsis originating from abdominal infections, urinary tract infections, or traumatic wounds causes indirect lung injury mediated by circulating inflammatory mediators, bacterial translocation, and remote organ crosstalk (54). Clinical multi-cohort studies further confirm that direct pulmonary and indirect extrapulmonary ARDS exhibit distinct molecular phenotypes and inflammatory pathway activation profiles, and that infection source is a key determinant of pathological network heterogeneity and clinical outcomes (55). Specifically, abdominal sepsis is associated with higher circulating endotoxin levels and more pronounced endothelial barrier dysfunction; urinary tract-derived sepsis often presents with milder initial lung injury but higher risk of secondary fungal superinfection; and trauma-induced sepsis additionally involves damage-associated molecular patterns released from injured tissues, which synergize with pathogen-associated molecular patterns to amplify inflammatory lung damage (56).

### 4.2 Dysregulation of the immune-inflammatory response

Within the pathological network, immune-inflammatory dysregulation acts as the core signal amplifier, receiving upstream stimulation from pathogens and mitochondrial damage, and in turn activating oxidative stress, coagulopathy, ferroptosis, and gut barrier disruption to propagate injury across the network.

In the context of sepsis, the host immune-inflammatory response becomes excessively activated and dysregulated, often resulting in multiple organ dysfunction syndrome. Patients with sepsis who develop acute lung injury exhibit a particularly poor prognosis, with mortality rates reported to reach approximately 33% (57). The pathological cascade involves the uncontrolled release of pro-inflammatory cytokines, damage to the pulmonary vascular endothelium, decreased alveolar surface tension, and the advancement of pulmonary interstitial fibrosis.

The inflammatory response in SA-ALI is characterized by a complex network involving multiple cell types and signaling pathways. A central effector unit, frequently referred to as the “inflammatory triangle”, comprises neutrophils, macrophages, and endothelial cells. Neutrophils, as primary innate immune responders, release NETs to capture and neutralize pathogens. NETs are web-like structures composed of decondensed DNA, citrullinated histones, and granular proteases released by activated neutrophils. Although NETs serve a protective function, excessive NET formation contributes directly to pulmonary vascular endothelial injury and microthrombosis, thereby exacerbating lung damage (58). In sepsis, NETs are released in pulmonary microvessels via the TLR4-Raf-MEK-ERK signaling axis; their histone and protease components directly induce alveolar epithelial and endothelial cell death, disrupt the alveolar-capillary barrier, and promote microvascular thrombosis, all of which are causally linked to ALI development (59). Clinical studies have also found that circulating MPO-DNA complexes, a biomarker of NET formation, are significantly elevated in SA-ALI patients and correlate with disease severity (60). Clinical studies have shown that increased plasma concentrations of NET components are associated with greater severity and higher mortality in patients with ARDS, highlighting the critical role of NETs as mediators of inflammatory injury (57, 61).

The imbalance in macrophage polarization significantly exacerbates the inflammatory response. Classically activated M1 macrophages produce substantial quantities of pro-inflammatory cytokines, such as IL-1, IL-6, and IL-8, which contribute to pronounced pulmonary inflammatory infiltration. Conversely, alternatively activated M2 macrophages, known for their anti-inflammatory and tissue-reparative roles, are frequently insufficiently activated during sepsis, resulting in an inability to effectively mitigate inflammation or facilitate tissue repair (62). While macrophages play a vital role in pathogen phagocytosis and the activation of adaptive immunity through antigen presentation, their dysregulated activation in sepsis perpetuates a deleterious hyperinflammatory state.

Endothelial cell injury constitutes a critical downstream consequence of this dysregulated inflammatory process. Prolonged inflammatory signaling leads to endothelial cell apoptosis and compromises the integrity of the vascular barrier. This disruption elevates the alveolar-arterial oxygen gradient and ultimately results in interstitial pulmonary edema. Therefore, endothelial dysfunction not only serves as a key indicator of deteriorating pulmonary function but also represents an essential therapeutic target for strategies aimed at vascular protection (63).

Active inflammation resolution pathways represent an equally critical host defense arm that is frequently dysregulated in severe sepsis. Specialized pro-resolving mediators (SPMs), including lipoxins, resolvins, protectins, and maresins are endogenously derived lipid mediators that orchestrate neutrophil clearance, macrophage efferocytosis, and tissue repair without impairing pathogen clearance (64). Impaired SPM biosynthesis in sepsis leads to persistent unresolved inflammation and delayed lung repair, and exogenous supplementation with resolvins or lipoxins has consistently shown protective efficacy in preclinical SA-ALI models (65). Clinical cohort studies further demonstrate that deficient SPM profiles in sepsis patients are associated with higher risk of ARDS development and worse clinical outcomes (66).

In terms of network crosstalk, excessive pro-inflammatory cytokines activate NADPH oxidase in endothelial and epithelial cells to drive ROS overproduction, forming the classic “oxidative stress-inflammation amplification cycle”. Activated neutrophils also release myeloperoxidase and free iron via NETs, which directly promote lipid peroxidation and ferroptosis in surrounding parenchymal cells (67). Furthermore, systemic inflammatory mediators disrupt intestinal tight junction integrity and perturb gut microbiota composition, initiating the gut-lung axis vicious cycle.

### 4.3 Oxidative stress injury and coagulation dysfunction

Oxidative stress and coagulation dysfunction form a tightly coupled effector module in the pathological network, downstream of both inflammatory activation and mitochondrial dysfunction, and in turn feedback to amplify immune dysregulation and ferroptosis.

Oxidative stress and coagulation dysfunction interact synergistically to establish a self-perpetuating vicious cycle that exacerbates ALI in the context of sepsis. Under normal physiological conditions, pulmonary homeostasis is preserved through a dynamic equilibrium between the production and clearance of oxygen free radicals (68). However, following microbial invasion, activated phagocytes and endothelial cells release a surge of inflammatory mediators, which stimulate macrophages and polymorphonuclear leukocytes to generate excessive ROS, thereby overwhelming the endogenous antioxidant defense systems (69).

This oxidative attack causes direct injury to lipids, proteins, and DNA present in alveolar epithelial and vascular endothelial cells, resulting in structural disruption and functional impairment. Moreover, ROS act as potent signaling molecules that trigger key inflammatory pathways such as nuclear factor kappa B (NF-κB) and drive the synthesis of pro-inflammatory cytokines including IL-1β, IL-6 and TNF-α. This process establishes a deleterious positive feedback mechanism, often referred to as the “oxidative stress-inflammation amplification” cycle, which perpetuates and intensifies tissue injury (70).

Coagulopathy represents a common and severe complication of sepsis, manifesting in approximately 50-70% of cases and closely associated with multi-organ failure (71). The activation of coagulation pathways occurs concomitantly with the inflammatory response. Systemic inflammation induces endothelial damage, exposing subendothelial collagen and triggering the extrinsic coagulation cascade. Simultaneously, inflammatory cells release pro-coagulant factors, such as platelet-activating factor, which facilitate platelet aggregation and microthrombus formation. This pro-thrombotic milieu is further exacerbated by the suppression of anticoagulant mechanisms, including reduced antithrombin levels and elevated plasminogen activator inhibitor-1 (PAI-1), culminating in an imbalance that favors coagulation over fibrinolysis (72).

The pulmonary consequences of these processes are profound. Extensive deposition of fibrin and proteins within the alveoli, combined with widespread pulmonary microthrombosis, leads to increased lung tissue density and fibrotic remodeling. This pathophysiological state results in ventilation-perfusion mismatch, intrapulmonary shunting, and impaired gas exchange capacity (73). The ensuing local ischemia and hypoxia accelerate parenchymal necrosis. Collectively, this intertwined pathophysiology underscores the potential importance of therapeutic strategies that concurrently target both antioxidant and anticoagulant pathways to disrupt this vicious cycle and attenuate lung injury.

At the network level, excessive ROS directly oxidizes membrane lipids to provide substrates for ferroptosis, and also activates the NF-κB pathway to sustain inflammatory activation, forming a bidirectional positive feedback loop with the immune-inflammatory module. Meanwhile, pulmonary microthrombosis causes local tissue hypoxia, which further exacerbates mitochondrial dysfunction and ROS generation, creating a hypoxic-oxidative stress vicious cycle at the tissue level (74).

### 4.4 Bidirectional regulation of the gut-lung axis

The gut-lung axis serves as the cross-organ amplification module of the pathological network, extending local pulmonary injury to systemic metabolic-immune disorders and feeding back to aggravate lung damage through circulating metabolites and bacterial products.

The gut-lung axis serves as a pivotal “cross-organ inflammatory regulatory axis” functioning as a significant amplifier of ALI in the context of sepsis. Contemporary studies substantiate the existence of bidirectional communication between the gut and lung at multiple levels, including their shared embryological origins, a unified mucosal immune system, and reciprocal functional interactions between these organs (75). The gut microbiota acts as a crucial “biological barrier” preserving intestinal homeostasis by competitively excluding pathogenic organisms and producing antimicrobial substances. However, during sepsis, factors such as splanchnic hypoperfusion, disruption of the intestinal barrier, and ischemia-reperfusion injury contribute to bacterial translocation. Microbes originating from the gut or their components, such as lipopolysaccharide (LPS), traverse the compromised mucosal barrier, enter systemic circulation, disrupt immune equilibrium, and provoke robust local and systemic inflammatory responses (76). Conversely, the systemic inflammatory cascade triggered by pulmonary infection, which is characterized by elevated IL-6 and TNF-α levels, exerts deleterious effects on the gut. This remote signaling perturbs microbial homeostasis, suppresses beneficial commensal populations, facilitates the proliferation of pathobionts, and further compromises the intestinal barrier. Consequently, a self-perpetuating, bidirectional vicious cycle emerges, described as “lung injury–gut dysbiosis–exacerbated lung injury” (77). This pathophysiological framework highlights that effective therapeutic interventions for sepsis-induced ALI must extend beyond the pulmonary system. Integrated strategies aimed at modulating the gut microbiota and preserving intestinal barrier integrity are imperative, thereby providing a compelling rationale for innovative “cross-organ collaborative therapies”.

Within the network framework, gut barrier disruption and bacterial translocation further activate systemic innate immunity and amplify the inflammatory storm, while reduced short-chain fatty acid production impairs macrophage antioxidant capacity and increases susceptibility to ferroptosis in the lung. This cross-organ regulatory loop explains why gut dysfunction is consistently associated with worse prognosis in SA-ALI patients (14).

### 4.5 Cross-talk between ferroptosis and cell death

Ferroptosis functions as the terminal effector module of the pathological network, integrating upstream signals from mitochondrial dysfunction, oxidative stress, and inflammation, and in turn releasing damage-associated molecular patterns (DAMPs) and free iron to further amplify inflammatory and oxidative injury.

Ferroptosis, an iron-dependent regulated form of cell death, plays a critical role in the pathogenesis of ALI during sepsis, exhibiting both unique and overlapping characteristics with other cell death modalities (78). It is primarily characterized by the uncontrolled accumulation of lipid peroxides, which leads to disruption of the plasma membrane, leakage of cellular contents, and ultimately cell death. Key regulatory mechanisms encompass lipid peroxidation, mitochondrial dysfunction, inhibition of GPX4, dysregulation of iron metabolism, and imbalances in systems such as the cystine/glutamate antiporter (System xc^-^) and p53 signaling pathways (46). Accumulating evidence from both animal models and patient biosamples verifies that ferroptotic death of alveolar epithelium and vascular endothelium accounts for a substantial proportion of tissue damage during sepsis, and pharmacological or genetic inhibition of ferroptosis consistently attenuates lung injury and improves survival in preclinical sepsis models (16). Mitochondrial dysfunction acts as a key upstream trigger of ferroptosis by producing excess ROS and disrupting intracellular iron homeostasis, forming a self-amplifying feedforward injury loop.

Mitochondria play a central role in the execution of ferroptosis. Dysregulation of mitochondrial autophagy (mitophagy), along with alterations in mitochondrial membrane potential, fusion, and fission dynamics, are closely linked to ferroptotic processes (79). Morphological changes include mitochondrial shrinkage, increased membrane density, loss of cristae, and elevated lipid peroxide accumulation, culminating in organelle and cellular demise (80). A distinctive feature of ferroptosis in sepsis-induced ALI is its complex interplay with other programmed cell death pathways, particularly autophagy. During the early stages of disease, moderate autophagy may confer protection by removing damaged organelles and inflammatory mediators. However, in later stages, excessive or dysregulated autophagy can degrade essential cellular components, paradoxically accelerating cell death and exacerbating lung injury (81).

Recent mechanistic investigations have elucidated specific molecular drivers underlying the process. Evidence presented by Li et al. indicates that the early growth response protein 1 (EGR1) directly binds to the promoter region of glutaminase 2 (GLS2), thereby transactivating its expression. This EGR1/GLS2 signaling axis facilitates ferroptosis and aggravates lung injury in experimental models of sepsis. Additionally, post-translational modification of EGR1 through arginine methylation enhances its protein stability, resulting in sustained upregulation (82). These findings highlight the EGR1/GLS2 pathway as a potential therapeutic target for the modulation of ferroptosis. Clinically, serum lactate, a well-recognized biomarker for tissue hypoperfusion and metabolic dysregulation in sepsis, has been shown to correlate with disease severity (83). Elevated lactate concentrations are associated with more severe pulmonary dysfunction, thereby linking systemic circulatory failure to the progression of lung injury and possibly to ferroptosis-related metabolic stress (84). Taken together, this body of evidence emphasizes the therapeutic potential of interventions aimed at key regulators of ferroptosis, such as EGR1 and GLS2, alongside strategies to restore metabolic equilibrium.

## 5 Innovative advances in therapeutic strategies

**Figure 3:**
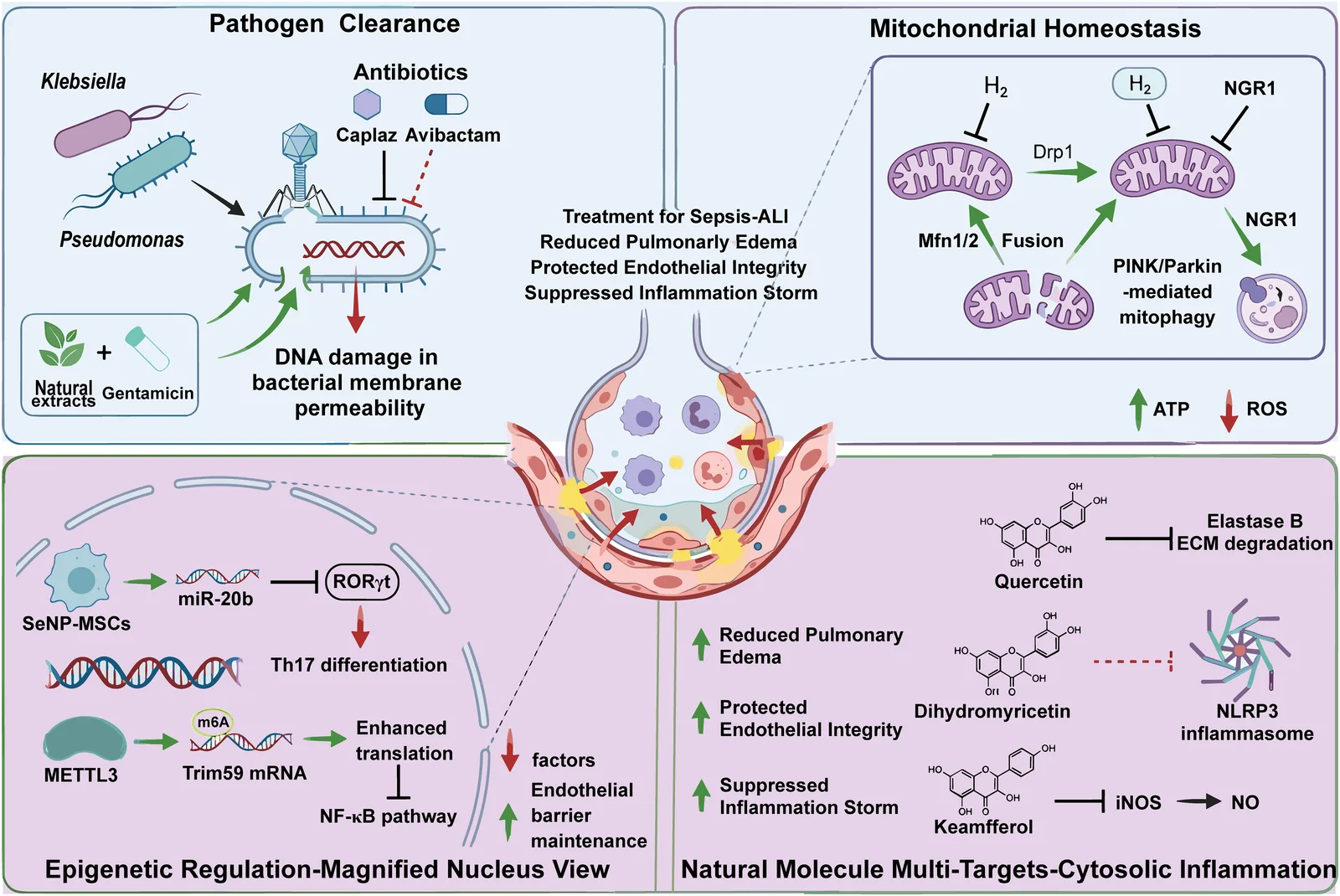
Schematic of integrated multi-stage, multi-target synergistic therapeutic strategies for sepsis-associated acute lung injury. This diagram establishes four complementary combinatorial therapeutic modules and elaborates their synergistic effects against the multi-node pathological network of SA-ALI:1) Pathogen clearance: Combination regimens of antibiotics, natural small-molecule extracts, and bacteriophages target bacterial membrane permeability and genomic damage to reverse multidrug resistance in Gram-negative pathogens.2) Mitochondrial homeostasis regulation: Hydrogen gas and notoginsenoside R1 balance mitochondrial fission (Drp1) and fusion (Mfn1/2), activate PINK/Parkin-dependent mitophagy, reduce ROS overproduction and restore cellular ATP to block the central injury hub.3) Epigenetic modulation: Selenium nanoparticle-loaded mesenchymal stem cells deliver miR-20b to suppress Th17 cell differentiation; METTL3-mediated m^6^A methylation stabilizes Trim59 mRNA to inhibit NF-κB cascades and relieve inflammatory storms at the transcriptional level.4) Multi-target natural compounds: Quercetin, dihydromyricetin, and kaempferol separately inhibit elastase B, NLRP3 inflammasome and iNOS signaling to alleviate pulmonary edema and preserve endothelial integrity. Combined administration of these four therapeutic branches simultaneously blocks multiple critical nodes across the pathological network, exerting superior lung-protective efficacy via coordinated upstream pathogen elimination, central mitochondrial protection and downstream inflammatory suppression compared with single-pathway monotherapies.

Guided by the multi-tiered pathological network framework, therapeutic research on SA-ALI has evolved from traditional single-target interventions to a multidimensional synergistic precision therapy system. The core innovative value of the therapies reviewed below lies in their layered targeting of the network: upstream pathogen clearance, central hub modulation, and downstream effector blockade. The core innovative value of the therapies reviewed in this section, including antibiotic optimization, phage therapy, mitochondrial-targeted intervention, epigenetic regulation, and natural small molecule application, is reflected in three major directions. The combined anti-infective strategy based on pathogen resistance mechanism and virulence factors, such as antibiotic-natural extract synergy and phage-antibiotic combination, which breaks through the drug resistance bottleneck by targeting bacterial membrane structure and inhibiting virulence factor expression. Another key direction is multi-target protection strategies rooted in pathological mechanism crosstalk, such as combining mitochondrial-targeted therapies (NGR1/H_2_) with ferroptosis inhibitors: this dual approach reduces ROS release at the source and blocks downstream lipid peroxidation, achieving dual blockade of both hub mechanisms and specific pathological pathways. Further progress comes from precise immune modulation via epigenetic regulation, including miR-20b-mediated inhibition of Th17 cell differentiation and METTL3/m6A/Trim59 axis-mediated endothelial barrier protection, which enables targeted immune-inflammatory regulation in place of non-specific broad-spectrum inhibition. At the same time, natural small molecule compounds, with the advantages of multi-target and low side effects, have become an important bridge connecting anti-infection, anti-inflammation and tissue damage repair, providing abundant candidate drugs for the clinical application of multi-strategy combination. The action targets and applicable courses of various therapies have clear temporal and spatial matching, laying a foundation for the subsequent formulation of phased synergistic treatment plans. (**Figure 3**).

### 5.1 Challenges and optimization of conventional antibiotic therapy

The advent of antibiotics markedly decreased mortality associated with infectious diseases; however, the rapid emergence of bacterial resistance now constitutes a significant global public health challenge. Both the overuse and misuse of antibiotics have facilitated the proliferation of multidrug-resistant organisms, thereby constraining therapeutic options and elevating mortality rates. Mechanisms contributing to antibiotic resistance include the activation of efflux pumps, alterations in drug target sites, and modifications in membrane permeability (15). Consequently, there is an urgent need to develop novel strategies for infection prevention and treatment.

In the 1990s, carbapenems served as the preferred therapeutic agents for the management of infections induced by *Klebsiella pneumoniae* producing extended-spectrum beta-lactamase (ESBL). However, widespread carbapenem use has led to the global emergence of carbapenem-resistant *Klebsiella pneumoniae* strains, with over 70% of resistance attributed to carbapenemase production (85). Currently, antibiotics such as cefpirome, cefotetan-tazobactam, and ceftazidime-avibactam are commonly employed. The fifth-generation cephalosporin cefpirome exhibits broad-spectrum activity against both Gram-negative bacteria (e.g., *Pseudomonas aeruginosa*, *Haemophilus influenzae*) and Gram-positive pathogens (e.g., methicillin-resistant *Staphylococcus aureus* [MRSA], *Salmonella*). It conquers MRSA resistance by targeting penicillin-binding proteins, which serve as essential enzymes in the synthesis of bacterial cell walls. Cefotetan-tazobactam combines the cell wall synthesis inhibition of cefotetan with the β-lactamase inhibitory effect of tazobactam, serving as a critical alternative to carbapenems, particularly for multidrug-resistant *Pseudomonas aeruginosa* infections (86). Additionally, the combination of amikacin and avibactam targets Gram-negative bacteria producing metallo-β-lactamases; avibactam irreversibly binds β-lactamases, thereby protecting amikacin from enzymatic degradation and substantially broadening its antimicrobial spectrum (15).

Beyond the development of novel antibiotics, synergistic approaches combining antibiotics with natural extracts offer promising avenues for combating drug-resistant bacteria. For instance, studies have demonstrated that co-administration of gentamicin with methanol extracts of Lactuca sativa disrupts the bacterial capsule, increases membrane permeability, facilitates gentamicin uptake, and enhances its DNA-damaging effects. This combination therapy achieved bacterial clearance rates two to three times higher than gentamicin monotherapy, while enabling reduced gentamicin dosages and lowering the risk of nephrotoxicity (87). Combination therapeutic regimens not only augment the effectiveness of existing antibiotics but also retard the development of antimicrobial resistance, thereby expanding the spectrum of clinical treatment options available.

Bacteriophages have also gained recognition as a viable alternative therapeutic approach. These viruses specifically infect, replicate within, and lyse bacterial cells and are classified into lytic and temperate phages. Lytic phages bind to specific receptors on the bacterial cell membrane, replicate intracellularly to generate progeny phages, and subsequently lyse the host cell. In contrast, temperate phages integrate their genetic material into the bacterial genome and replicate concomitantly with bacterial cell division. Lytic phages are generally preferred due to their capacity for rapid and selective eradication of pathogenic bacteria while sparing commensal microbiota. Beyond their direct bacteriolytic activity, phages can synergize with the host immune system by modulating inflammatory responses and enhancing phagocytic activity (88, 89). Additional advantages of phage therapy include their ability to penetrate bacterial membranes, favorable safety and toxicity profiles, and the potential to modify bacterial structures to attenuate virulence. Phage therapy predominantly targets three Gram-negative pathogens: *Klebsiella pneumoniae*, *Pseudomonas aeruginosa*, and *Acinetobacter baumannii* (90). The phenomenon of phage-antibiotic synergy arises when both agents are administered concurrently, leveraging their distinct bactericidal mechanisms to achieve superior efficacy compared to monotherapy (90). Consequently, phage therapy represents a promising strategy for the management of bacterial infections.

Currently, phage therapy for multidrug-resistant Gram-negative pulmonary infections is mostly at the preclinical and compassionate use stage. Phage-antibiotic combination regimens exert synergistic bactericidal effects by disrupting bacterial envelope integrity, reversing efflux pump-mediated resistance, and reducing resistance mutation rates, and have shown favorable efficacy against *K. pneumoniae*, *P. aeruginosa*, and *A. baumannii* in both animal models and preliminary clinical studies (90).

Phage therapy predominantly targets three critical Gram-negative pathogens in pulmonary infections: *Klebsiella pneumoniae*, *Pseudomonas aeruginosa*, and *Acinetobacter baumannii*. The phenomenon of phage-antibiotic synergy arises when both agents are administered concurrently, leveraging their distinct bactericidal mechanisms to achieve superior efficacy compared to monotherapy: combination regimens reduce the probability of bacterial resistance emergence, and antibiotics can disrupt bacterial biofilms and outer membranes to further enhance phage infectivity. Clinical experience from compassionate use cases and early-phase studies confirms that inhaled phage formulations achieve high local concentrations in the respiratory tract, making them particularly suitable for ventilator-associated pneumonia and chronic pulmonary infection settings (91). Nevertheless, clinical translation remains constrained by key limitations: the narrow host range of natural phages requires strain-specific susceptibility matching prior to clinical use, bacteria may develop phage resistance via receptor mutation or endogenous immune defense systems, and large-scale confirmatory randomized controlled trials as well as standardized manufacturing and regulatory frameworks are still lacking (90). Consequently, phage therapy represents a promising strategy for the management of drug-resistant bacterial pulmonary infections.

### 5.2 Mitochondria-targeted therapeutic approaches

A substantial body of research has established that mitochondrial dysfunction is a central factor in the pathogenesis of sepsis, wherein excessive inflammatory mediators and stress-induced cellular damage disrupt mitochondrial dynamics. Mitochondria, essential organelles for human cellular function, are involved not only in energy metabolism but also in critical biological processes such as signal transduction, cell differentiation, cell cycle regulation, and programmed cell death. Mitochondrial dysfunction represents an early pathogenic event in sepsis, featured by impaired oxidative phosphorylation, excessive reactive oxygen species accumulation, and disrupted mitochondrial fission-fusion balance, which directly damages alveolar epithelial and pulmonary microvascular endothelial cells and amplifies systemic inflammatory cascades (92). Clinical cohort studies further confirm that circulating mitochondrial damage biomarkers such as plasma mitochondrial DNA are elevated prior to overt organ failure, and are independently associated with disease severity and 28-day mortality in septic patients (93). Extensive evidence suggests that enhancing mitochondrial function and modulating oxidative stress confer cytoprotective effects (45).

Hydrogen gas, recognized as a novel antioxidant, exhibits distinct therapeutic potential in the context of sepsis-associated acute lung injury. Zhao et al. developed a rat model of severe sepsis to evaluate the effects of varying hydrogen concentrations (2% and 60%) (45). Their findings indicated that both concentrations mitigated acute lung injury, augmented antioxidant enzyme activities in lung tissue and serum, and decreased levels of oxidative products and proinflammatory cytokines. Notably, the higher concentration of hydrogen demonstrated superior efficacy. The underlying mechanisms include the reduction of mitochondrial fragmentation and restoration of mitochondrial cristae integrity via anti-inflammatory and anti-apoptotic actions, modulation of signaling pathways, activation of mitochondrial fusion proteins such as mitofusin 1 and 2 (Mfn1/2), and inhibition of mitochondrial fission protein dynamin-related protein 1 (Drp1) expression. Concurrently, hydrogen administration elevated the enzymatic activities of superoxide dismutase (SOD) and glutathione peroxidase (GSH-Px) in lung tissue, lowered malondialdehyde (MDA) levels, attenuated oxidative stress-induced damage, and exerted an adjunctive therapeutic effect (45).

Active constituents derived from traditional Chinese medicine also represent promising candidates for mitochondria-targeted interventions. Hou et al. investigated Notoginsenoside R1 (NGR1), the principal bioactive compound of Panax notoginseng, in septic murine models (94). Their results demonstrated that NGR1 administration significantly decreased mitochondrial fragmentation in lung tissue, increased ATP production by 40% to 50%, and substantially ameliorated lung injury parameters, including alveolar hemorrhage and inflammatory infiltration. Mechanistic analyses revealed that NGR1 directly interacts with the GTPase domain of Drp1, inhibiting its activity and preventing its translocation to the mitochondrial membrane, thereby suppressing mitochondrial fission. Additionally, NGR1 upregulated the expression of mitochondrial autophagy-related proteins such as Parkin and PINK1, facilitating the removal of damaged mitochondria and further enhancing mitochondrial function. This study not only identifies Drp1 as a viable therapeutic target but also provides empirical support for the contemporary application of active compounds from traditional Chinese medicine in mitochondrial therapy (94).

Regarding clinical translation, hydrogen inhalation has demonstrated favorable safety and preliminary therapeutic potential in early-phase clinical studies for acute respiratory failure, with ongoing randomized controlled trials further evaluating its efficacy on mortality outcomes in sepsis-associated ARDS (95). In contrast, notoginsenoside R1 (NGR1) and other traditional Chinese medicine monomers remain confined to preclinical *in vitro* and animal studies, with no published clinical trial data available for sepsis-associated acute lung injury to date (96).

The main limitations of mitochondria-targeted therapies include the challenge of maintaining stable alveolar hydrogen concentrations during spontaneous breathing or mechanical ventilation, which requires standardized FiH_2_ delivery systems to ensure consistent therapeutic tissue levels (97); for NGR1, extremely low oral bioavailability (a BCS Class III drug with poor membrane permeability) necessitates nanocarrier or modified delivery systems to achieve sufficient *in vivo* drug exposure and target tissue distribution (98).

From a network synergy perspective, combining mitochondria-targeted agents with ferroptosis inhibitors achieves a dual-blockade effect: it suppresses mitochondrial ROS generation at the upstream source and blocks downstream lipid peroxidation cascades, and this multi-node intervention strategy has been shown to exert significantly stronger pulmonary protective efficacy than monotherapy in preclinical acute lung injury models (16).

### 5.3 Novel targets for epigenetic intervention

Epigenetic regulation dynamically modulates gene expression through mechanisms such as non-coding RNA regulation, histone modifications, and DNA methylation. It plays a pivotal role in inflammatory responses and apoptosis during acute lung injury in sepsis. Its reversible nature makes it an ideal therapeutic target, offering new avenues for intervention. Clinical studies reveal significantly elevated Th17 cell levels within the bronchoalveolar lavage fluid of patients affected by sepsis-related acute lung injury, positively correlated with disease severity (99). RORγt, a specific transcription factor for Th17 cells, directly participates in their differentiation (100). Inhibiting RORγt reduces Th17 cell differentiation, thereby exerting anti-inflammatory effects. Building on this, Zhang et al. developed chitosan-functionalized selenium nanoparticles. After co-incubating these nanoparticles with bone marrow mesenchymal stem cells (BMSCs), they observed that the nanoparticles entered cells via BMSC phagocytosis, releasing selenium to activate selenoproteins (e.g., glutathione peroxidase) while simultaneously upregulating miR-20b expression. miR-20b directly binds the 3’-UTR region of RORγt, inhibiting its mRNA translation and thereby reducing Th17 cell differentiation (99). Furthermore, this combined approach reduces reactive oxygen species levels in lung tissue, mitigating oxidative stress damage (99). This study not only validates the feasibility of “nanocarrier-stem cell” interventions but also establishes a novel paradigm for integrating epigenetic regulation with cell therapy.

Endothelial cell injury represents the terminal stage of acute lung injury in sepsis, and preserving endothelial integrity contributes to sepsis improvement. Maintaining endothelial function is governed by numerous genetic and epigenetic regulatory mechanisms, with m6A methylation playing a pivotal role in this process (101). M6A methylation exerts its function by modulating RNA splicing, subcellular localization, stability and translation efficiency. METTL3 serves as a core methyltransferase in m6A modification, and it enhances mRNA translation efficiency through catalyzing the formation of m6A methylation modifications. Analysis of clinical samples has revealed a significant downregulation of METTL3 expression in the lung tissues of sepsis patients. This reduced METTL3 expression is negatively correlated with the levels of endothelial barrier damage markers, such as vascular endothelial cadherin (VE-cadherin). Mechanistic studies revealed that METTL3 enhances translation efficiency by methylating mRNA of endothelial protection-related genes (e.g., Trim59). Trim59 further suppresses NF-κB pathway activation, thereby reducing endothelial cell apoptosis and inflammatory cytokine release to maintain endothelial barrier integrity. Exogenous supplementation of METTL3 significantly improved pulmonary edema, hypoxemia, and survival rates in septic mice, suggesting that the METTL3/m6A/Trim59 axis may represent a novel therapeutic target for acute lung injury in sepsis (102).

All epigenetic interventions for SA-ALI are currently in preclinical cellular and animal studies, with no clinical trials initiated (103, 104). Related miRNA-based agents are in phase I trials for autoimmune diseases, but their safety and efficacy in critically ill septic patients remain untested (105). Core translational challenges include low *in vivo* targeted delivery efficiency, susceptibility to nuclease degradation, and the genome-wide off-target effects of m^6^A modulation (105, 106). For clinical application, precise timing of administration is critical: intervention during the peak inflammatory phase may avoid excessive immunosuppression and impaired pathogen clearance (107).

### 5.4 Therapeutic potential of natural small molecules

Natural compounds offer several distinct advantages over synthetic pharmaceuticals. Primarily, they tend to elicit fewer adverse effects. Furthermore, they serve as a valuable reservoir of bioactive agents. Importantly, multiple natural compounds can modulate diverse signaling pathways implicated in acute lung injury associated with sepsis via multi-targeted mechanisms. These attributes underscore the potential of natural small molecules as alternative therapeutic options.

Evidence from recent studies identifies elastase B as a key extracellular virulence factor secreted by *Pseudomonas aeruginosa*. This enzyme degrades the host extracellular matrix, facilitating bacterial invasion and dissemination, while simultaneously triggering inflammatory responses and oxidative stress that exacerbate infection severity (108). Quercetin has been shown to form stable complexes with elastase B through intermolecular interactions, thereby inhibiting its synthesis and enzymatic activity, which contributes to the attenuation of inflammation-induced tissue damage. Additionally, kaempferol reduces nitric oxide concentrations in pulmonary tissue by downregulating nitric oxide synthase expression, leading to decreased nitric oxide production (109). Kaempferol also exerts anti-inflammatory effects, mitigating cellular pulmonary edema and reducing apoptosis (110). Moreover, dihydroquercetin alleviates acute lung injury in sepsis by inhibiting NLRP3 inflammasome-mediated apoptotic pathways (111). Collectively, these findings highlight the promising therapeutic potential of natural small-molecule compounds in managing sepsis-induced acute lung injury.

Most natural small molecules for sepsis-associated acute lung injury remain in the preclinical stage of cellular and animal studies, with no approved clinical indications to date. While early-phase clinical trials of quercetin are ongoing in surgical populations to evaluate its anti-inflammatory and organ-protective effects, no published phase IIa trial data are available to confirm its efficacy for perioperative acute lung injury prophylaxis (112, 113). Core limitations of these agents include low target specificity, moderate therapeutic potency, and unfavorable pharmacokinetic properties, with low pulmonary tissue concentrations after systemic administration being a key barrier to *in vivo* efficacy (113, 114). From a network pharmacology perspective, their greatest clinical value lies in inherent multi-target characteristics: they simultaneously modulate bacterial virulence, inflammatory cascades, and oxidative stress nodes, making them promising adjuvant agents that can enhance therapeutic efficacy and reduce toxicity when combined with antibiotics or mitochondria-targeted therapies (112, 114).

### 5.5 Technologically advanced therapeutic approaches

In recent years, the development of multi-omics technology and organ chip models has provided a technology-driven new paradigm for analyzing the heterogeneity of SA-ALI and breaking through the limitations of traditional research (115). These advanced technologies provide essential tools for deconstructing the heterogeneity of the pathological network across different patient populations and disease stages, and for screening node-specific therapeutic agents (116, 117). The integrated analysis of single-cell transcriptome, epigenome and metabolome not only clarifies the subgroup heterogeneity of alveolar epithelial cells, vascular endothelial cells and immune cells, but also excavates the core module of “metabolism-immunity” cross-regulation of the gut-lung axis, providing a high-throughput means for screening pathogen-specific and course-specific molecular markers. Emerging technological platforms including multi-omics-guided precision medicine, lung-on-a-chip disease models, and gene-edited cell therapies provide new paradigms for SA-ALI drug development and personalized treatment. Multi-omics stratification enables identification of molecular subphenotypes with distinct pathological features, allowing matching of patients to mechanism-appropriate therapies (118). Physiomimetic lung-on-a-chip systems that recapitulate the human alveolar-capillary barrier and respiratory mechanics provide a human-relevant high-throughput platform for drug efficacy and toxicity screening, bridging the translational gap between animal studies and clinical trials (117). Humanized lung organoids and lung-intestinal chip models break through the limitations of cross-species differences and multi-organ interaction simulation of traditional animal models, can accurately reproduce the lung injury process induced by pathogens and the bidirectional regulation of the gut-lung axis, and provide a research platform closer to human physiology for high-throughput screening of new targeted drugs. In addition, stem cell/nanocarrier-mediated targeted delivery technology effectively solves the problem of low *in vivo* delivery efficiency of epigenetic regulation, ferroptosis inhibition and other therapies, and becomes a key technical support connecting basic mechanism research and clinical transformation. The deep integration of cutting-edge technology and SA-ALI pathological mechanism research has promoted the transformation of this field from “phenomenon description” to “precise molecular analysis”, providing technical guarantee for the realization of individualized treatment (99, 119).

### 5.6 Heterogeneity of special populations and comorbidities

The heterogeneity of SA-ALI patients in clinical practice is not only reflected in pathogens and disease courses, but also originates from the superimposed regulation of physiological characteristics of special populations and basic comorbidities, which is also a key uncovered field for the transformation from basic research to clinical practice. From a network perspective, comorbidities and special physiological states reshape the topology of the pathological network, altering the activity of core hubs and the strength of crosstalk between modules, which means universal therapeutic regimens cannot be applied uniformly. SA-ALI exhibits pronounced heterogeneity in pathological networks and therapeutic responses across special populations including older adults, immunocompromised patients, and pediatric cohorts. Older adults display age-related impairment of mitochondrial quality control and dysregulated innate immune activation characterized by blunted early inflammatory responses and prolonged immunosuppression, which renders them more susceptible to secondary infections and severe lung injury, alongside distinct optimal therapeutic windows compared with younger patients (120). Children have immature intestinal flora and imperfect lung tissue development, so the cross-organ injury mediated by the gut-lung axis is more significant, and the pathogen susceptibility and injury degree have age dependence (121, 122). Immunocompromised patients frequently present with opportunistic pathogen infections and dysregulated inflammatory responses, requiring adjusted antimicrobial and immunomodulatory regimens. At the same time, basic comorbidities such as diabetes, chronic obstructive pulmonary disease, and cardiovascular disease form superimposed injury with SA-ALI by aggravating oxidative stress, destroying epithelial barrier and regulating immune metabolism (123). For example, the hyperglycemic state of diabetes can amplify mitochondrial ROS release and ferroptosis, and the chronic injury of airway epithelium in COPD patients can significantly increase the probability of pathogen invasion and amplify pulmonary inflammatory response (92, 124). The reconstruction of SA-ALI pathological network under the background of special populations and comorbidities reveals the core connotation of “individualized treatment”, and also puts forward new requirements for clinical formulation of hierarchical intervention strategies, that is, it is necessary to adaptively adjust the core therapeutic targets and intervention intensity in combination with patients’ physiological characteristics and basic diseases (125).

## 6 Discussion

Acute lung injury in sepsis, as one of the most common severe complications of sepsis, has long troubled the field of critical care medicine due to its high incidence, high mortality rate, and complex pathophysiological characteristics. In recent years, advances in basic medical and clinical research have expanded understanding beyond the traditional ‘pathogen infection-tissue injury’ model towards a comprehensive perspective encompassing ‘pathogen-host interactions-multisystem network dysregulation’. Nevertheless, clinical management continues to face significant challenges, with no breakthrough progress achieved thus far.

This review systematically combs the complete pathological network of SA-ALI from pathogen invasion to tissue injury, as well as the recent progress in targeted therapy. The core innovation lies in breaking through the traditional research framework of “single pathogen/single mechanism/single therapy” and constructing an integrated research system of “pathogen typing-course dynamics-mechanism cross-precision therapy”. This network-driven perspective brings forward three key conceptual advances that are not systematically elaborated in conventional reviews of sepsis-associated acute lung injury. First, it explains the persistent failure of single-target anti-inflammatory or antioxidant therapies in clinical trials: the highly interconnected pathological network is equipped with redundant compensatory pathways that can bypass a single blocked node, thus blunting the efficacy of isolated interventions (126, 127). Second, it identifies that targeting central regulatory hubs such as mitochondria delivers superior therapeutic efficacy compared with isolated intervention on downstream effector pathways, as hub nodes simultaneously modulate multiple downstream pathological cascades (92). Third, it demonstrates that optimal treatment requires sequential multi-target combinations tailored to the dynamic activation state of the pathological network across different disease stages, rather than uniform regimens applied throughout the entire disease course (128). Clarifying the common and specific mechanisms of lung injury induced by different pathogens, revealing the cascade amplification rule of multiple pathological mechanisms centered on mitochondrial dysfunction, proposing a multi-target synergistic therapeutic strategy based on mechanistic crosstalk, and integrating cutting-edge technologies with clinical heterogeneity demands to point out the core direction for the subsequent research in this field. At present, there are still many urgent core scientific problems to be solved in SA-ALI research: how to screen the specific molecular markers of SA-ALI induced by different pathogens and apply them to clinical typing; whether the upstream core regulatory pathway of ferroptosis as a cell death cascade switch has pathogen and population specificity; the key metabolites in the gut-lung axis metabolic regulatory network that can be used as cross-organ protection targets; how to construct an efficient targeted delivery system to realize the clinical transformation of epigenetic regulation, ferroptosis inhibition and other therapies; the reconstruction law of SA-ALI immune metabolic network and individualized intervention strategy under the background of special populations and comorbidities.

To address these limitations and clinical challenges, future research should advance along core strategic directions. Multi-omics technologies, including single-cell transcriptomics, epigenomics and metabolomics, should be integrated to map dynamic molecular profiles across different disease stages. This integration can help identify specific diagnostic biomarkers and staging targets, thereby providing a solid foundation for personalised therapies. Leverage organ-on-a-chip and organoid technologies to establish humanised sepsis-induced lung injury models, simulating the human physiological microenvironment and disease heterogeneity to enhance the efficiency of translating basic research into clinical applications. Explore sequential and synergistic therapeutic strategies based on the pathological characteristics of different disease phases (inflammatory activation, injury progression, and repair), formulating a phased collaborative approach of “anti-infection –anti-inflammation-repair ‘phased synergistic protocols to achieve a precise equilibrium between’ infection control and host protection”.

The essence of acute lung injury in sepsis lies in “self-injury” caused by immune-metabolic imbalance. Its treatment hinges on disrupting the vicious cycle of pathogen invasion, multi-mechanism amplification and tissue damage, aiming to achieve a precise balance between infection control and immune homeostasis. Research and application in pathogen-host interactions, cross-organ regulation, epigenetic mechanisms, multi-omics technologies, targeted delivery systems, and advanced disease models, alongside next-generation personalised, multi-targeted integrated treatment models, will improve outcomes for patients with septic lung injury. This approach enables precise prevention, control, and effective treatment of this critical condition.
